# The Effectiveness of Electrical Acupuncture Stimulation in Reducing Levels of Self-reported Anxiety of Lung Cancer Patients during Palliative Care: A Pilot Study

**Published:** 2017-08

**Authors:** Yin-Qing HU, Yi-Fan WU, Li-Li HOU

**Affiliations:** Dept. of Nursing, Shanghai Pulmonary Hospital, Tongji University, Shanghai, China

**Keywords:** Lung cancer, Palliative care, Electrical acupuncture stimulation, Self-reported anxiety, Patients

## Abstract

**Background:**

Lung cancer is a serious threat to human health and life worldwide. Anxiety is common amongst palliative care patients with lung cancer and adversely affects quality of life. Acupuncture is an effective and safe treatment method used for the treatment of depressive mood status. We aimed to assess the influence of electrical acupuncture stimulation on self-reported anxiety in palliative care among patients with lung cancer.

**Methods:**

This pilot study had an experimental, 3-group, research plan. A total of 188 participants were enrolled from the Shanghai Pulmonary Hospital, Tongji University, Shanghai, China from 2014–2015. This pilot study had an experimental, 3-group, research plan. In TEAS group, participants received standardized palliative care and electrical acupuncture stimulation in Zusanli, Sanyinjiao and Hegu acu-points. Group MS received standardized palliative care and muscle stimulation nearby nonacupoint. Controlled group received standardized palliative care. The patients maintained their assigned acupuncture stimulation for 7 days. Demographic Instrument, Karnofsky Performance Scale Index, SF-16 health questionnaire and Self-Rating Anxiety Scale (SAS) were used.

**Results:**

The mean SAS scores in TEAS Group before and after electrical intervention in palliative care intervention were 31.17±7.55,34.58±13.98 and 27.86±6.73, (*P*=0.00) QoL score showed elevation from 57.13 in 8th day to 60.12 in 28th day, rising further to 5%. Comfort Score showed continuous elevation trend for 28 days.

**Conclusion:**

Electric acupuncture stimulation could reduce the anxiety of patients, promote rehabilitation and increase the quality of life among patients with lung cancer in palliative care.

## Introduction

Lung cancer has become the most common cancer worldwide, accounting for 17% of new cancer cases and 23% of the death cases (1). Lung cancer is a serious threat to human health and life worldwide. Cancer accounted for 13% of all deaths in 2008 (WHO, 2014a) (2). The morbidity and mortality elevated sharply in the developing world. In China, lung cancer has now become the leading cause of cancer deaths in both urban as well as rural areas (3).

Many lung cancer patients continue to receive treatment, i.e. surgery or radiotherapy, in the hope of curing their disease. However, the majority of them experience severe physical and psychological distress dying in the process of curative treatment at hospitals. Their families usually suffer great emotional, social, and spiritual torment (4). Lung cancer patients experience both spiritual distress and physical pain before death. Therefore, effective palliative care intervention plays a vital role in ensuring the physical and psychological well-being of lung cancer patients as well as supporting the needs their families. Moreover, it also helps in improving the patients’ quality of life to make their terminal life more comfortable, dignified, and meaningful (5). Furthermore, psychological care and support also play an essential role in palliative care (6). Almost all lung cancer patients have negative emotions, such as depression and anxiety. French (7) supported that psychological therapy could improve the quality of life for cancer patients. It is normal for lung cancer patients to experience fear, distress, and anxiety; but it is necessary to provide them psychological counseling and help them engage in meaningful activities to improve their quality of life at the end stages of their lives (6).

Anxiety is common amongst palliative care patients and affects adversely quality of life (6). Relax therapy, anti-anxiety drugs and other medications could be arranged for patients. Apart from the above approaches, acupuncture is an effective and safe treatment method that has been used for the treatment of depressive mood status (8). Acupuncture is an ancient medical treatment, which was originated in China. Acupuncture is used to treat several symptoms and conditions related to cancer and its treatment side effects. There are a few studies demonstrated acupuncture to be an effective and safe complementary therapy for cancer care (6–8).

The concepts of yin and yang have been expanded on from Taoism and have long been thought to influence a person’s health and illness. Illness is a result of an imbalance between yin and yang (9). The forces of yin and yang formulate Chi. According to traditional Chinese medicine, excessive or suppressed emotions affect the normal circulation of Chi in the body. Stimulating acupoints in the body generates and smoothens the flow of Chi (10). In the past few years, complementary therapies such as acupressure, acupuncture, and transcutaneous electrical acupoint stimulation are effective for managing depression or anxiety symptoms (11). Acupuncture might be superior to antidepressants in improving clinical response and reducing depressive symptoms of PSD patients (12). Transcutaneous electrical acupoint stimulation improved depressive mood status among elders in a nursing home (7). No study has specifically applied electrical acupuncture stimulation to treat anxious mood status of patients with lung cancer during palliative care. There is a paucity of information with regard to the effectiveness of management of symptoms indicating anxiety in palliative care of patient.

The present study was aimed to assess the influence of electrical acupuncture stimulation on self-reported anxiety in palliative care among patients with lung cancer.

## Materials and Methods

This pilot study had an experimental, 3-group, research plan. All the patients suffered from non-small cell lung cancer with stages III or IV. Non-small cell lung cancer is defined using accepted diagnostic criteria based on clinical assessment, molecular analyses of tumor biopsy specimens and computed tomography of the chest. The participants who were less than 18 yr-old, with impaired cognitive function, other secondary tumors, heart, cerebral or renal failure, and loss of follow-up or without the sufficient clinical database were excluded. Informed consent was obtained from respondents in the study. The ethics committee of the Shanghai Pulmonary Hospital, China (K14-170) approved the study protocol for retrospective analysis.

A total of 188 participants were recruited from Shanghai Pulmonary Hospital, Tongji University, Shanghai, China. Eligible participants were randomly divided into three groups from December 2014 to June 2015. Randomization was conducted using a computer-generated allocation list by an assigned researcher who was not involved in the intervention and assessment. In TEAS (transcutaneous electrical acupuncture stimulation) group, participates received standardized palliative care and electrical acupuncture stimulation, of Zusanli, Sanyinjiao and Hegu acupoints. Group MS received standardized palliative care and muscle stimulation nearby non acupoint.

**Fig.1:**
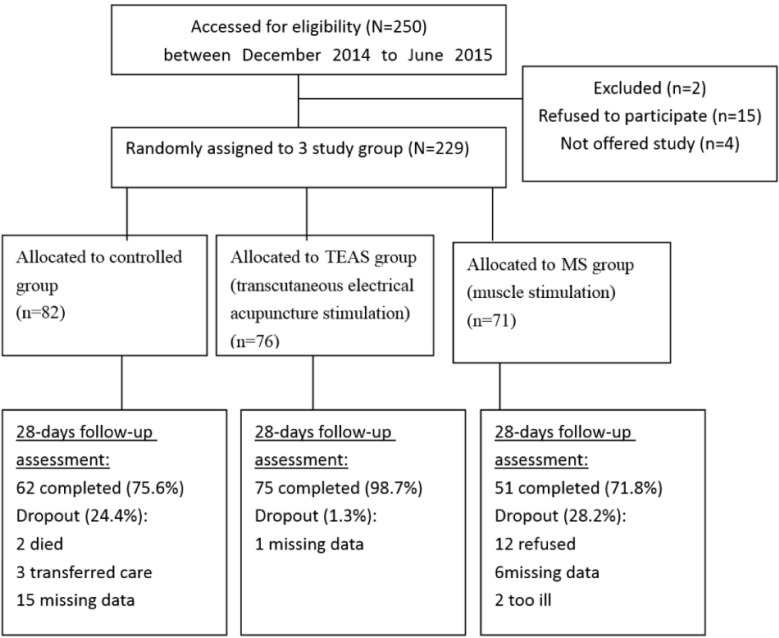
Flow diagram of this study

**Fig. 2:**
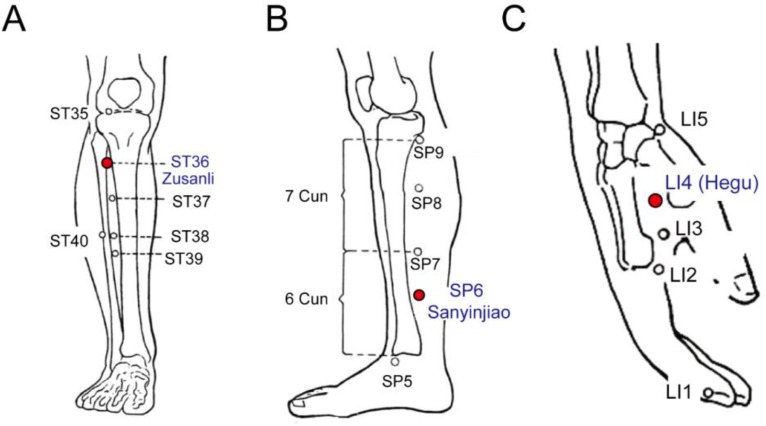
Location of acupuncture: A: Zusanli (ST36); B: Sanyinjiao (SP36); C: Hegu (LI4). The red solid points in A, B, and C represent Zusanli, Sanyinjiao, and Hegu (LI4), respectively

Controlled group received standardized palliative care (Fig. 1). In the experimental group, 4 trained registered nurses, who had received same training courses in Chinese medicine, applied electrical acupuncture stimulation. To control the validity and reliability of the electrical acupuncture stimulation procedure, 3 acupoints were designated to improve anxious mood status: Zusanli (ST36), Sanyinjiao (SP6) and Hegu (L14) (Fig. 2).

The patients maintained their assigned acupuncture stimulation for 7 days. The surface electrical stimulation devices and application parameter were as follow: G6805-II pulse acupuncture treatment instrument from Shanghai Medical Technology Company, Ltd (Shanghai, China); sparse-dense wave stimulation (sparse wave, 30Hz; dense wave, 100Hz) with an intensity of 6 to 15 V; and the corresponding parts of the body experienced a slight quiver, with a duration of 20 minutes each time.Demographic Instrument, Karnofsky Performance Scale Index, SF-16 health questionnaire and Self-Rating Anxiety Scale (SAS) were used in this study.

Data analysis was performed using SPSS 16.0 for Windows (SPSS Inc., Chicago, IL). *P*-value of 0.05 was set as the level of significance. Descriptive statistics were used to summarize all variables. To evaluate the differences in the demographic information and anxiety scores between the 3 groups, F- test was performed; the Chi-square test was applied if the dependent variables were normal or ordinal variables.

## Results

As shown in demographic characteristics Table 1, there were no significant differences between the 3 groups with age (*P*=0.91), sex (*P*=0.29), financial status (*P*=0.41), family history (*P* =0.09), BMI (*P*=0.21), temperature (*P*=0.24), heart rate (*P*=0.18) and respiratory rate (*P*=0.39). Blood pressure (*P*=0.20 and 0.06) and KPS scores (*P*=0.251) were also not statistically significant between these 3 groups.

**Table 1:** Demographic characteristics (N=188) and comparison in 3 groups

**Variables**	**Controlled Group(n=62)**	**MS Group (n=75)**	**TEAS Group (n=51)**		
	**n (%)**	**n (%)**	**n (%)**	**X2** or **F test**	***P***
Sex				2.42	0.29
Male	51(82.3%)	60(80%)	36(70.6%)		
Female	11(17.7%)	15(20%)	15(29.4%)		
Financial				1.78	0.41
status					
Pension	45(72.6%)	50(66.7%)	31(60.8%)		
Non-pension	17(27.4%)	25(33.3%)	20(39.2%)		
Family History				4.91	0.09
Yes	1(1.6%)	1(1.3%)	4(7.8%)		
No	61(98.4%)	74(98.7%)	47(92.2%)		
Age	58.81±10.50	59.45±8.20	59.98±8.51	0.09	0.91
BMI	24.05±2.76	22.97±4.52	23.59±2.72	1.59	0.21
Body temperature	36.81±0.29	36.73±0.32	36.79±0.31	1.43	0.24
Heart rate	81.52±4.52	82.68±5.76	83.57±7.32	1.75	0.18
Respiratory rate	20.98±11.54	19.61±1.15	21.37±7.21	0.96	0.39
Systolic	125.65±15.33	125.41±15.25	121.00±15.25	1.63	0.20
Pressure					
Diastolic	78.48±7.47	78.89±9.71	75.33±8.56	2.82	0.06
Pressure					
KPS	92.54±6.76	91.76±7.58	88.46±6.89	1.41	0.25

The Table 2 shows statistical significant for the mean SAS scores in TEAS Group before and after electrical intervention during palliative care intervention. On the other hand, there were no significant differences of SAS scores in MS Group before and after intervention.

**Table 2:** Comparison of SAS scores in 3 groups (n=188)

**Variable**	**1 day before**	**8th day**	**28th day**
Controlled Group(n=62)	34.28±8.39	41.84±13.01	35.63±9.92
MS Group (n=75)	35.37±14.74	37.85±15.92	35.53±14.29
TEAS Group (n=51)	31.17±7.55	34.58±13.98	27.86±6.73
F test	2.02	3.61	7.01
*P*	0.11	0.06	< 0.001

**Fig. 3:**
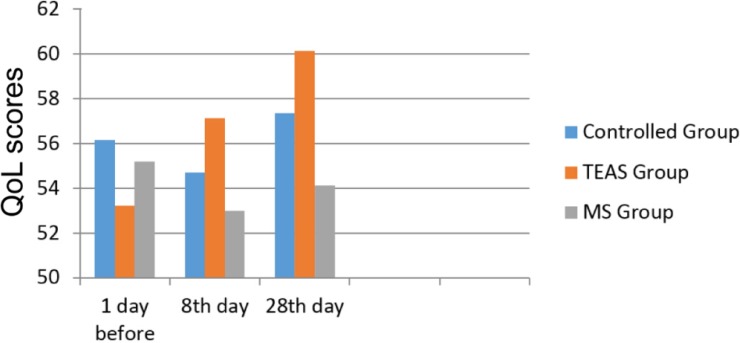
Qol scores of the three different groups. Qol scores of the three different groups at 1 day before, 8^th^ day, and 28^th^ day, respectively

**Fig. 4:**
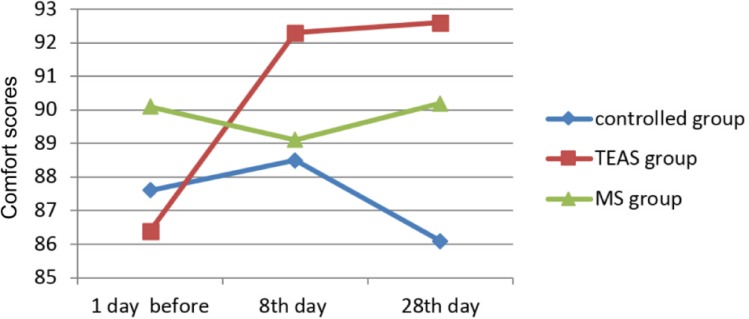
Comfort scores of the three different groups. Comfort scores of the three different groups at 1 day before, 8^th^ day, and 28^th^ day, respectively

Furthermore, there were no significant differences of SAS scores in controlled group during palliative care. Fig. 3 demonstrated QoL scores between 3 groups during intervention. QoL score showed elevation from 57.13 in 8th day to 60.12 in 28th day, rising further to 5%. Comfort Score showed continuous elevation trend for 28 days (Fig. 4).

## Discussion

The results of this study supported the application of electrical acupuncture stimulation so as to improve mood status of patients in palliative care. Mood disorders like anxiety or depression is one of spleen-deficiency syndromes. Further spleen-deficiency syndrome could be treated by tonifying spleen by stimulation of specific acupoints as per traditional Chinese medicine (13). SP 6 in Spleen Meridian and ST 36 in Stomach Meridian are usually targeted in acupuncture therapy to improve spleen-deficiency syndrome, according to the meridian theory (14). In terms of pathogenesis, anxiety is closely related to stagnation of liver-Chi. Hegu (L14) points were used in acupuncture treatment for the regulation of liver as well as for the activation of mental activity (15). Chinese people believed that Chi is the universal vital energy that is inherent in each person until death. The flow of Chi in the body affects bodily constitution and health. Electrical acupuncture stimulation could be applied not only to relax the human body but also to stimulate the channels governing the flow of Chi. When Chi increases, harmony reached between yin and yang. As such, electrical acupuncture stimulation could balance chi and thus promoted quality of life and regulated anxiety levels during operation (16). Acupressure plays a key role in both contemporary and alternative medicine (17). According to the meridian theory of Chinese medicine, energy Chi flows through meridians, which are invisible circuitries or energy channels in the body. Acupressure corrects the flow of Chi by application of pressure (18).

The study tested the effectiveness of electro acupuncture to decrease depression using electrical stimulation of the nerve cells around acupoints. There were significant improvements in mood status of participants (19). Electrical acupuncture stimulation was effective in the management of the syndrome of depression in hemodialysis patients (20). The plenty of recent evidences suggested that acupuncture when combined with antidepressant therapy has a faster therapeutic onset rate than pharmacotherapy alone (21). Five clinical studies showed significant improvement in anxiety and depression over time in patients who underwent acupuncture treatment, from which a pilot study had stated feasibility of administering acupuncture as alternative palliative therapy for advanced cancer patients. Their results demonstrated psychological distress, life satisfaction and had higher positive scores of mood states during acupuncture (20). At the same time, acupuncture is useful for reducing the severity of anxiety. The rate of serious anxiety decreased from 15.6% to 2.2% before and after acupuncture, respectively (22). There was a 57.8% reduction in the severity of anxiety in all of the patients (23). The effect of acupuncture on symptoms such as nausea, vomiting and pain as well as anxiety and the mood status of cancer patients were evaluated and reported to be useful in an earlier report (23).

Electrical acupuncture stimulation is a safe, easy, noninvasive intervention to manage the anxiety mood and improve the quality of life among lung cancer patients in palliative care. It helps to treat the symptoms of lung cancer and promote the comfort. Furthermore, it is a comprehensive oncologic service for patients with terminal stage lung cancer for the improvement of their quality of life. It facilitates the efficient allocation of medical recourse, which is especially necessary for anxious surgical patients. At the same time, nurses apply electrical acupuncture stimulation to patients; human contact and touch or oral health education are provided to satisfy needs of anxious patients. Therefore, applying electrical acupuncture stimulation to surgical patients might increase contact and touch with patients.

However, there are some limitations in this study. First, it is unclear whether these findings could be generalized to other kinds of cancer patients in palliative care. Secondly, clinical data in the present study was collected in the form of paper; therefore, it is possible that other clinical factors (treatment, diagnosis time, medicine) could have influenced the effect of electrical acupuncture stimulation. Thirdly, it is a polite study, which has small sample size and study on large sample size is recommenced for future studies.

## Conclusion

Electric acupuncture stimulation could reduce the anxiety of patients, promote rehabilitation and increase the quality of life among patients with lung cancer in palliative care. The results could serve as a reference in the treatment of anxiety among advanced lung cancer patients.

## Ethical considerations

Ethical issues (Including plagiarism, informed consent, misconduct, data fabrication and/or falsification, double publication and/or submission, redundancy, etc.) have been completely observed by the authors.
